# The impact of mobile point of care interaction on the September asthma epidemic: a randomized pilot study

**DOI:** 10.1186/1710-1492-8-S1-A28

**Published:** 2012-11-02

**Authors:** Scott Cameron, Shauna Filuk, Jo-Anne St Vincent, Allan Becker

**Affiliations:** 1 Division of Pediatric Allergy and Immunology, The University of Manitoba, Winnipeg, Manitoba, Canada

## Objective

To prevent asthma exacerbations during the fall asthma epidemic utilizing weekly short text messaging focusing on asthma symptoms and control.

## Methods

A prospective pilot study of text messages sent to asthma patients between the ages of 11-21 was performed. The control group received texts focusing on healthy living and activity. Recruitment and the intervention occurred in the summer and fall of 2011. Asthma Control Test (ACT) scores and the number of asthma exacerbations requiring physician intervention were primary outcomes. Secondary outcomes included SMS response rates and times. Qualitative outcomes included patient and caregiver satisfaction with the intervention.

## Results

Seventeen patients participated, although three did not complete the study. The average age was 14.9 ± 2.5 (range 12-20). ACT scores were significantly better in the intervention group than in the control group at the end of September (21.3 ± 2.1 vs 16.0 ± 5.0 p < 0.05). Exacerbations were significantly more frequent in the control group than the intervention group (5 vs 0, p = 0.009). Text response rates were more frequent and quick in those receiving the intervention versus controls (78% response rate with a 12 minute average vs 58% with a 266 minute average). Participants and primary caregivers believed the intervention improved communication with their medical team.

## Conclusion

There was significant improvement in asthma control and exacerbations with the use of weekly short text messages about asthma symptoms and asthma control in this pilot study. Further investigation of this easy to implement, inexpensive, and teen approved intervention’s utility in improving asthma management is certainly warranted.

